# Effects of Scaffold Pore Morphologies on Glucose Transport Limitations in Hollow Fibre Membrane Bioreactor for Bone Tissue Engineering: Experiments and Numerical Modelling

**DOI:** 10.3390/membranes11040257

**Published:** 2021-04-02

**Authors:** Shuai Wang, Hazwani Suhaimi, Mostafa Mabrouk, Stella Georgiadou, John P. Ward, Diganta B. Das

**Affiliations:** 1 Department of Chemical Engineering, Loughborough University, Loughborough LE113TU, UK; ws910219@sina.com (S.W.); hazwani.suhaimi@ubd.edu.bn (H.S.); mostafamabrouk.nrc@gmail.com (M.M.); S.Georgiadou@lboro.ac.uk (S.G.); 2 Refractories, Ceramics and Building Materials Department, National Research Centre, 33El Bohouth St. (former EL Tahrir St.), Dokki, Giza P.O. Box 12622, Egypt; 3 Department of Mathematical Sciences, Loughborough University, Loughborough LE113TU, UK; john.ward@lboro.ac.uk

**Keywords:** glucose diffusion, mathematical modelling, hollow fibre membrane bioreactor, tissue engineering, pore microstructure

## Abstract

In the current research, three electrospun polycaprolactone (PCL) scaffolds with different pore morphology induced by changing the electrospinning parameters, spinning time and rate, have been prepared in order to provide a fundamental understanding on the effects pore morphology have on nutrient transport behaviour in hollow fibre membrane bioreactor (HFMB). After determining the porosity of the scaffolds, they were investigated for glucose diffusivity using cell culture media (CCM) and distilled water in a diffusion cell at 37 °C. The scanning electron microscope (SEM) images of the microstructure of the scaffolds were analysed further using ImageJ software to determine the porosity and glucose diffusivity. A Krogh cylinder model was used to determine the glucose transport profile with dimensionless variables within the HFMB. The paper discusses the roles of various dimensionless numbers (e.g., Péclet and Damköhler numbers) and non-dimensional groups of variables (e.g., non-dimensional fibre radius) on determining glucose concentration profiles, especially in the scaffold region. A negative linear relationship between glucose diffusivities across PCL scaffolds and the minimum glucose concentrations (i.e., concentration on the outer fibre edge on the outlet side (at z = 1 and r = 3.2) was also found. It was shown that the efficiency of glucose consumption improves with scaffolds of higher diffusivities. The results of this study are expected to help in optimizing designs of HFMB as well as carry out more accurate up scaling analyses for the bioreactor.

## 1. Introduction

Due to the shortage of bone grafts and the likelihood of disease transmission via the grafts from other patients, bone tissue engineering (BTE) has become an important approach for replacing bone tissue defects in recent years [1,2]. Due to the stringent requirements of cell density and mechanical strength in bone grafts, many factors need to be considered to achieve a successful bone tissue engineered product. In this regard, one of the key factors is to have a porous scaffold with an inter-connected network of pores that could be used as a replacement for the extra cellular space (ECS) in TE bioreactors. This can then function as a template for cell support, infiltration, and proliferation [3]. Whilst the cells construct new bone tissues in the bioreactors, the scaffolds are resorbed or degraded [4]. However, one of the challenges that still exists in this procedure relates to the diffusion limitation of nutrients (e.g., glucose), implying that the further the cells in the ECS are from the nutrient source, the more deficient the cells will be in the nutrient concentration. Typically, nutrient concentration at any particular position is inversely proportional to √x, where x is the distance of the cells from the source of the nutrient. In the literature, there have been a significant amount of discussion on the necessity of overcoming this nutrient limitation for optimum cell viability within the porous scaffold in TE bioreactors [5,6].

Amongst the engineering approaches that have been explored to solve these problems is the fabrication of hollow fibre membrane bioreactors [5,7,8], referred to as HFMBs in this paper. HFMBs are broadly utilized as a system that separates various media (e.g., cell culture media and waste products). As a module, HFMB are stuffed together with a number of hollow capillaries to frame a group, which is then positioned in a shell and sealed at the two ends. The shell-and-tube like structure induce or prevent transportation of the components of each medium. Cells are grown and supported on the scaffolding material where layers of hollow fibre membranes are present. These membranes are permeable to only certain solutes, depending on the pore size of the membrane. The solutes are typically nutrients (e.g., glucose and oxygen) and waste products (e.g., lactic acid). The nutrients diffuse across the membrane where cells feed on them while the waste products produced diffuse back into the membrane and out of the system. This constitutes the circulation system, which mimics the native capillary network found in vivo. These bioreactors have been developed to enhance the delivery of nutrients as well as other molecules (e.g., oxygen and protein) to the cells within the ECS, which permits volume increment of the obtained tissue [5,7,8]. The waste products (e.g., lactic acid) are removed through the network of hollow capillaries placed within these systems.

**Figure 1:**
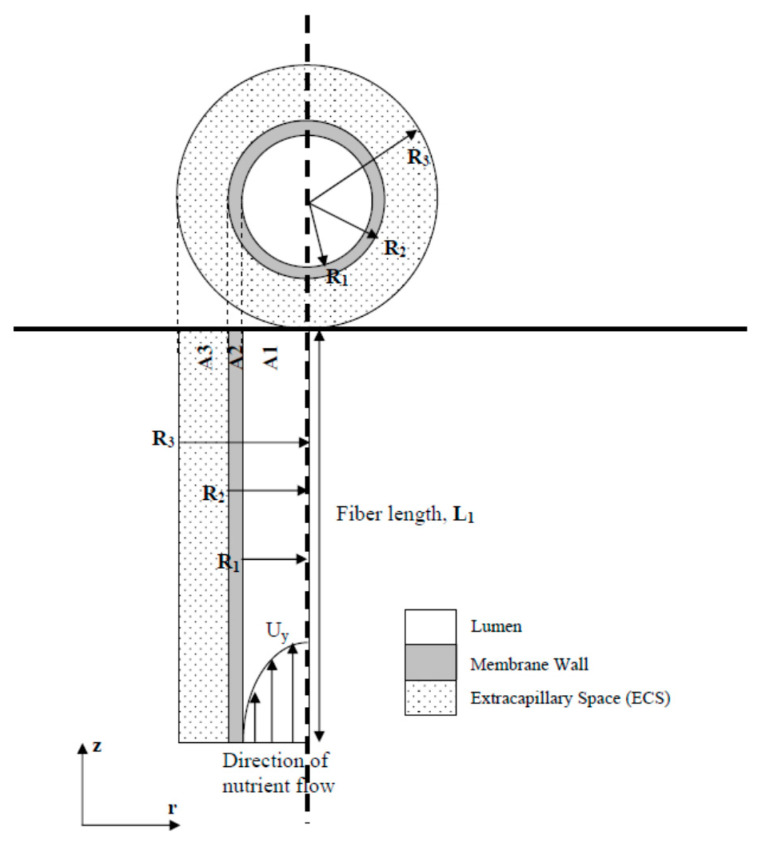
Schematic diagram of a hollow fibre membrane bioreactor (HFMB). Three domains are defined, namely, A1—fibre lumen, A2—membrane wall, and A3—extracapillary space (ECS). Please refer to Table 2 for the dimensions of the respective sections and other key parameters. The figure is reproduced from [5] with copyright permission.

It should also be noted here that the HFMB is a perfusion-type bioreactor, which implies that the nutrient transport to the cells is governed by the convection and diffusion of the nutrients in the membrane lumen (hollow capillaries) and diffusion of the nutrients in cell culture media (CCM) through the lumen membrane and the ECS. A schematic diagram that represents the HFMB is presented in Figure 1. It has been reported in earlier publications (e.g., [5]) that the nutrient transport can be enhanced within this type of bioreactor. In addition, it has been observed that high cell density can be achieved in HFMB, (e.g., see [9]), which suggests that a mammalian cell density of the order of 10^8^ cells/mL may be achieved in HFMB, which is close to the cell density in bone tissue. While these studies point toward the success of HFMB as a system for growing bone tissues, there are still shortcomings in our fundamental understanding in the behaviour of nutrients, oxygen as well as waste product transport through the HFMB. Therefore, these systems should be subjected to continuous in-depth investigations in order to determine their true diffusion performance [10,11,12].

To determine the concentration of small molecules such as glucose, many technologies have been developed such as electrochemical techniques [13], fluorescence spectroscopy [14], and nuclear magnetic resonance microscopy (NMR) [15]. However, these methods cannot directly monitor the diffusion process continuously in situ due to the small size of the HFMB. Therefore, a number of mathematical modelling approaches have been reported to determine a method that can simulate the HFMB performance and avoid the potential of infection/damage to the cells from in situ measurements [16].

A simple and facile diffusion cell was introduced by Suhaimi et al. [17] to measure the nutrient diffusivity in scaffold ex situ, which enabled the determination of glucose diffusivity across porous materials in water and cell culture media (CCM) in conditions similar to those expected in HFMB. However, we believe that the exact quantification of these diffusion processes remains incomplete due to a lack of quantification on how the membrane and scaffold microstructures affect the nutrient transport behaviour in the bioreactor. It has been reported earlier that image processing tools can be used effectively to analyse images of pore microstructures of the membranes in order to quantify important factors such as porosity, tortuosity, and diffusion coefficient [18], which affect the nutrient transport behaviour. Various computational modelling frameworks have also been developed to generate the glucose concentration profiles inside the HFMBs using assumed values. However, combinations of these approaches have not employed experimentally deduced glucose diffusivities in TE membranes and scaffolds imbibed with the cell culture media (CCM). In other words, most of the existing models found in the literature are based on the assumption that the glucose diffusivities in the culture medium are similar to the ones in water and all microstructures have similar effects. However, Suhaimi et al. [17,18,19] determined experimentally deduced glucose diffusivities in cell-free and cell-seeded TE membranes and scaffolds. With recent advances in the understanding of glucose diffusivity in cell culture medium, computational modelling, porous material preparation (e.g., electrospinning techniques), and image processing approaches, it should be possible to address these issues effectively. We therefore aim to address this point in this paper by presenting a framework for incorporating the effects of scaffold pore morphologies on glucose transport in HFMB. 

Taking these into consideration, herein, three porous polycaprolactone (PCL) scaffolds were prepared by manipulating electrospinning parameters that include dissolved polymer feeding rate and time. The objective of this task was simply to create different porous microstructures to fulfil the aim of the paper. Scanning electron microscope (SEM) images were recorded for these scaffolds and were further processed using ImageJ software in order to determine the pore morphology characteristics for relating these to the glucose diffusivity data. Glucose diffusivities in both water and cell culture media (CCM) imbibed electrospun fibres were also determined at 37 °C. Properties of the HF membrane wall (Figure 1) were defined the same in all cases. In addition, the effective glucose diffusion coefficients obtained from image processing were used to create the glucose concentration profiles through the PCLs’ HFMB. Scaling up of such systems could be attained by changing the dimensionless groups of parameter effects on the glucose concentration profiles, which is also discussed in this paper. 

## 2. Materials and Methods

Although this work aimed to develop an image-based simulation method with a view to capture the effects of membrane pore morphology on nutrient transport in HFMB, it is important to calibrate the image processing method. Keeping this in mind, the work involved a number of experiments as discussed below.

PCL scaffolds were fabricated in the current research work using an electrospinning technique with different feed flow rate and time with the objective to create different pore microstructures. Poly(ε-caprolactone) (PCL) (MW = 80,000 daltons), dichloromethane (DCM) (cat. no. 270997) and N, N-dimethylformamide (DMF) (cat. no. 227056) were purchased from Sigma-Aldrich (Dorset, England). Dulbecco’s modified Eagle medium (DMEM) (Life Technologies Ltd., Paisley, UK) was used as the cell culture media (CCM). The CCM typically contains inorganic salts (e.g., potassium chloride and sodium chloride), amino acids (e.g., glycine), vitamins (e.g., folic acid and riboflavin), and other components (e.g., phenol red and sodium pyruvate). Distilled water was employed as a reference fluid in this work.

### 2.1. Fabrication of Porous Poly(ε-caprolactone) (PCL) Scaffolds

To synthesize the PCL scaffolds, first, PCL (12% *w*/*v*) was dissolved in DCM and DMF (3:1, *v*/*v*) as a co-solvent system. The solution was homogeneously mixed by agitating with a magnetic stirrer at 600 rpm for at least 2 h at room temperature (25 ± 1 °C) prior to the electrospinning process. For the process of electrospinning, the solution of PCL was placed in one and/or two 3 mL plastic syringes fitted with hollow flat metal needle with a tip diameter of 0.56 mm. The voltage was applied by a high voltage power supply (Series FC, Glassman High Voltage Inc.). The voltage supply was fixed at 15 kV at a distance of 12 cm between a needle and collector and it was maintained throughout the electrospinning process. The electrospun fibres were collected on a 10 × 13 cm^2^ flat aluminium plate. A syringe pump (model 11 plus, Harvard Apparatus, USA) was used to control the polymer flow rate for the electrospinning. The experiment was carried out in a sealed environment at 25 ± 1 °C (room temperature) and at 45% relative humidity. Three different samples were prepared in this study with different experimental time and flow rate to develop different microstructural properties. The duration of electrospinning was 1.5 h (with one syringe) or 45 min (with two syringes). The feed flow rate was set at 1 or 2 mL/h. The experiments with 1 mL/h and 1.5 h were repeated three times under the same conditions in order to confirm the reproducibility and reliability of this design for the fabrication of the scaffolds. The processing parameters are shown in Table 1.

It should be noted that when we used two syringes in this work (sample 3), the same polymer (PCL) solution was used in an attempt to create a different set of fibre characteristics by depositing the electrospun fibres at different rates in the electrospun fibre collector. All other experimental conditions such as the imposed voltage in the system were kept the same. In principle, a range of other experimental possibilities exist that may provide different properties of the electrospun fibres such as the use of different polymer solution, distance between tip and collector, or different voltage.

### 2.2. Characterization of Scaffolds and Membranes

#### 2.2.1. Determination of Fibre–Fibre Space and Fibre Diameter of Electrospun Scaffold

Scanning electron microscopy (Carl Zeiss field emission gun (FEG)-SEM LEO 1530VP), which can provide images of the electrospun sample microstructures by scanning them with a focused beam of electrons, was used in the measurement of fibre–fibre distance and fibre diameter for all porous membranes/scaffolds prepared in this work. The operating principles of SEM are found elsewhere [20,21] and, therefore, are not discussed in this paper. Due to the non-conductivity of the polymers, PCL samples were provided with a 2–20 nm thin coating of electrically conducting metal, namely, gold/palladium (Au/Pd) using a sputter coating machine (Emitech, SC7640). Then, the coated samples were monitored using the SEM. Both the top and cross-sectional views of the samples were taken. Fibre–fibre distance and fibre diameter were measured after setting the scale by the reference distance on the SEM images. For each image, one hundred fibres were randomly chosen for the measurements. The mean value, distribution of fibre–fibre distance and fibre diameter were then determined.

#### 2.2.2. Determination of Porosity (∅) and Tortuosity (τ) of Scaffold and Membrane

In TE, both membranes and scaffolds consist of multilayers of staggered and overlapping networks of fibres. These kinds of structures are hard to image directly in SEM images. Importantly, from a purely physico-chemical point of view, this results in increased deviations of the glucose diffusion paths (tortuosity) from straight line paths, which in turn increases the diffusional distance and resistance. Therefore, to represent the effective changes in the significance in diffusion due to changes in microstructure in a porous media, the porosity and tortuosity need to be taken into account. According to the literature, porosity is defined as the ratio of voids volume to total volume and was calculated from Equation (1).

(1) $$\emptyset =1-\frac{V_{m}}{V_{t}}$$

where *V_m_* is the volume of solid space in scaffold and *V_t_* is the total volume of the scaffold (length × width × thickness). In this study, a pycnometric method was employed in the laboratory to determine the porosity, as it provides the basic concepts for understanding and due to its low cost. According to this method, porosity was calculated from Equation (2).

(2) $$\emptyset =1-\frac{m_{1}+m_{2}-m_{3}}{V_{t}\rho_{w}}$$

where *m*_1_ is the mass of dry porous sample; *m*_2_ is the mass of pure water at pycnometer level, *m*_3_ is the mass of porous sample saturated in pycnometer levelled with water; and *ρ_w_* is the water density, which is defined as 0.9970 g/cm^3^ at room temperature. For the porosity measurement, the scaffolds were assumed to be fully soaked (soaking in water for 4, 8, and 12 h) effectively in water in the pycnometer. Furthermore, the mean porosity values were estimated and used in the tortuosity calculation. Unlike porosity, which quantifies the nutrient transfer path reduced in a cross-sectional area, tortuosity is a way to describe the complexity of a porous diffusing pathway [22].

Tortuosity (*τ*) is defined by the increase in the diffusion distance that results from irregular pore distribution including bending and curves. In this study, tortuosity values were determined by Equation (3).

(3) $$D_{e}=\frac{D\emptyset }{\tau }$$

where *D_e_* is the effective diffusion coefficient through the pore space of porous scaffold; *D* is the self-diffusion coefficient (assumed to be isotropic) in CCM filling the pore; ∅ is the isotropic porosity of membrane/scaffold media; and *τ* is the isotropic tortuosity. In this study, the diffusion coefficient of glucose (*D*) in CCM was considered as a constant with a value of 9.58 × 10^−10^ m^2^/s at 37 °C in water as previously reported [18,19].

#### 2.2.3. Determination of Effective Glucose Diffusivity in Membranes and Scaffolds

**Figure 2:**
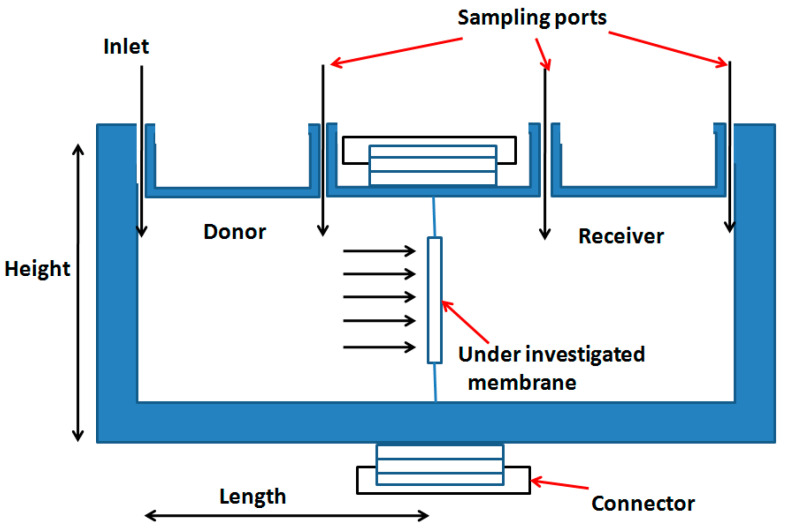
Schematic diagram of the diffusion cell used in the measurements of glucose diffusivity in commercial membranes and in-house electrospun scaffold materials.

Glucose diffusion coefficients in membranes and scaffolds were measured experimentally ex situ using a diffusion cell, as illustrated in Figure 2, using both distilled water and CCM. The change in glucose concentration in both water and CCM was measured by an YSI analyser (YSI 2300 STAT Plus^TM^ Glucose and Lactate Analyser, Life Science, England).

All electrospun scaffolds were pre-treated before starting the experiments through soaking in deionized water for overnight to ensure the complete wetting condition for PCL scaffolds [22]. The receptor phase was filled with either CCM or water, and the donor phase was loaded with glucose solution. Before the solution was fed into the chambers, pure water/CCM and glucose solution were both placed in a water bath to be preheated for at least 1 h to equilibrate at either 27 or 37 °C. A thermostated water bath was employed for the total duration of the experiments in order to maintain the experimental temperature condition. At each time period, a sample of 25 μL of glucose solution was withdrawn for the analysis of glucose concentration. In this situation, the consumption could be ignored as there are no changes in the volume and composition in both chambers (phases). For CCM, the whole experiment period was about 7–11 h depending on when the contamination would take place, but for the water, it was up to 24 h as no contamination was expected, and the glucose diffusion coefficients were then calculated based on Equation (4):

(4) $$V_{d}\frac{\partial C_{d}}{\partial t}=-D_{e}A\frac{C_{d}-C_{r}}{l}$$

where *V_d_* is the volume of solution in the donor phase, and *C_d_* and *C_r_* are the concentrations of glucose in the donor and receptor phase, respectively. The interval time is coded as *t*, the diffusion coefficient as *D_e_*, the area of the membrane through which the solution diffuses through is *A*, and l is the thickness of the membrane.

### 2.3. Image Processing

A well-known Java language software ImageJ [23] was utilized to process all SEM images of the pore microstructure. This software has programming options through macros, which permit new calculations and embedding modules containing new assets for the processing of image and computerized investigation. In this work, this analysis was conducted to describe the porosity, tortuosity, and glucose transport through different scaffolds, as discussed below.

#### 2.3.1. Determination and Calibration of Scaffold Porosity

Multiple images of the electrospun scaffolds and membranes were read using the “imread” function one at a time. These images were then converted to grayscale images using the “rgb2gray” function. All SEM images were converted to black and white images using the function “im2bw” for further processing. This was done by selecting a certain threshold value from the function “graythrash”, which was used as a reference for the conversion. Afterward, the images were filtered for unnecessary noises by removing all connected components below 50 pixels using the function “bwareaopen”. In addition, a negative of the image was recorded. Hence, the black part in the image signifies the scaffolds fibres, while the white part in the images relates to the pores. After completely filtering the images, the properties such as the area and perimeter of the image were extracted using the function “regionprops”. Using the area property, the porosity of the image was calculated by dividing the area of pores with the complete area of the image.

Moreover, the calibration process for the images was done at two levels; the first level of calibration was done by choosing the threshold value for the black and white conversion of the image. Instead of utilizing the optimum level of conversion, a threshold value of conversion was obtained by using the obtained data for different scaffolds from our previous work [18] to get the porosity value equal to their experimental results. The calibration was done using the basic curve fitting method. The optimum threshold was plotted against the found threshold (for which the porosity is equal to experimental porosity) and a straight line was fitted, which was extrapolated to calculate the threshold level for the rest of the scaffolds (using the equation of line). From this calibration, the final porosity was found to be closer to the value obtained from the experiments.

#### 2.3.2. Determination of Tortuosity of the Prepared Scaffolds

A set of assumptions was made to calculate the tortuosity of the prepared scaffold in this work. First, the topmost layer of scaffold obtained from SEM was used for which the shortest possible path (straight line) was considered. Second, the transport of glucose through the scaffold pores was assumed (represented as white part of the image) wherever they were present. In the absence of connected pores, glucose was assumed to move along the circumference of the scaffold fibres, taking into account that there might be multiple fibres clubbed together. Therefore, the distance between the start and the end of the clustered fibre (along the path of straight line) was obtained from the processed image, which was divided with fibre diameter and rounded off to the next closest integer (as all fibres are not of equal diameter). In addition to the above assumptions, the effective distance between two points at the opposite side of the image was calculated, which was divided by the straight-line distance to calculate the tortuosity value.

#### 2.3.3. Determination and Calibration of Diffusivity of Electrospun Fibres in Cell Culture Media (CCM) and Water from Image Processing

First, the calibration was done in two steps which involved calibrating the values using straight line fit. In particular, the experimental diffusion coefficient was plotted against the calculated diffusion coefficient from the in-house experimental setup (Figure 2) obtained from Equation (2) in which a straight line was fitted. From the equation of the straight line, all diffusion coefficients were evaluated. The second step was to calibrate these values further for higher accuracy. Experimental values were divided by calibrated values, which were plotted against the calibrated values and fitted with a second order curve. To obtain the final value, the calibrated value was multiplied by the corresponding value from the obtained curve.

The calibration of glucose diffusivities in water was done using the CCM results obtained from the above calibration. The calibration in any other media can be done in exactly the same way using the obtained results in CCM media as a reference. Effective diffusivity in water was obtained from Equation (2). This value was divided by the experimental value in water and plotted against the finally calibrated value divided by the effective diffusivity in CCM media (from Equation (2)), a linear curve fitting was used. The final value in water was obtained by multiplying effective diffusivity in water with the corresponding value from the straight line.

### 2.4. Bioreactor Modelling

**Figure 3:**
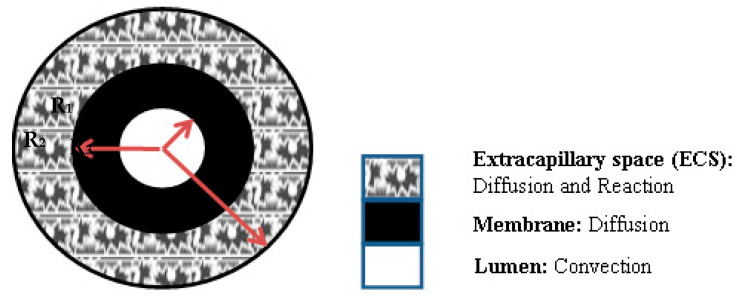
A single hollow fibre defined as a Krogh cylinder indicating the dominant nutrient transport mechanism.

To analyse the overall glucose transport behaviour through the microporous scaffold within the HFMB, a mathematical model was used to predict the glucose concentration profile. The Krogh cylinder model [24,25] was used where HFMB is defined of consisting of numerous parallel-arranged hollow fibres. The hollow fibres in each section are responsible for the nutrient supply to a cylindrical surrounding section. The interstitial space between the fibres is ignored for the purpose of modelling as we consider a single hollow fibre in this paper. Figure 3 shows a schematic diagram of a single hollow fibre (Krogh cylinder). Fibre lumen is from r = 0 to r = R_1_, where it is filled with cell culture media to supply the nutrients to the cells. Membrane wall is from r = R_1_ to r = R_2_, across which, the nutrient (e.g., glucose diffuses to the ECS region). From r = R_2_ to r = R_3_ is the ECS region, where the cells grow and proliferate on the scaffolds.

#### 2.4.1. Glucose Transport Equations

As shown in Figure 1 and Figure 2, fibre lumen region (R_1_) is dominated by laminar flow with a steady fluid (CCM) velocity described by the continuity equation for conservation of fluid mass, Stokes’ law for conservation of fluid momentum, and convection–diffusion equation for the conservation of glucose mass. The flow of CCM is continuous laminar flow with relatively low Reynolds number ($Re=\frac{U_{0}a}{ʋ}$), therefore, it is assumed that the flow in lumen is Newtonian incompressible fluid and has a Poiseuille laminar flow (Equations (5)–(7)). On each of the membrane interfaces, continuity of concentration (c) and diffusive flux (−D∂c/∂r)) are imposed. All the symbols and dimensional values used in the simulations can be found in Table 2.

(5) $$2U_{0}\left(1-r^{2}\right)\frac{\partial c}{\partial z}=D_{l}\left(\frac{1}{r}\frac{\partial }{\partial r}\left(r\frac{\partial c}{\partial r}\right)+\frac{\partial^{2}c}{\partial z^{2}}\right)\;\;\;\;\;\;\;\;\;\;\;\;\;\;\;\;\;\;\;\;\;\;\;0<r<a$$

with the boundary conditions:

(6) $$c=C_{0}\;\;\;\;\;\mathrm{at}\;z=0$$

(7) $${D_{l}\frac{\partial c}{\partial r}|}_{a^{-}}={D_{m}\frac{\partial c}{\partial r}|}_{a^{+}}\;{c|}_{a^{-}}={c|}_{a^{+}}\;\mathrm{at}\;r\;=\;a$$

where ${|}_{a^{-}}$ represents the limit near lumen-membrane boundary in the lumen side (r<a); ${|}_{a^{+}}$ represents the limit near lumen-membrane boundary in the membrane side (a<r). In the membrane region, due to the semi-permeability of membrane and the glucose concentration difference, glucose transport is only governed by diffusion process across membrane (Equations (8) and (9)).

(8) $$\;D_{m}\left(\frac{1}{r}\frac{\partial }{\partial r}\left(r\frac{\partial c}{\partial r}\right)+\frac{\partial^{2}c}{\partial z^{2}}\right)=0,\;\;\;\;\;\;\;\;\left(a<r<a+m\right)$$

with the boundary conditions:

(9) $${D_{m}\frac{\partial c}{\partial r}|}_{a+m^{-}}={D_{m}\frac{\partial c}{\partial r}|}_{a+m^{+}}\;{c|}_{a+m^{-}}={c|}_{a+m^{+}}\;\mathrm{at}\;r\;=\;a\;+\;m$$

where ${|}_{a+m^{-}}$ represents limit near membrane-ECS boundary in the membrane side (r<a+m); ${|}_{a+m^{+}}$ represents the limit near membrane-ECS boundary in the ECS side (a+m<r). In the ECS region, the radial advection ignored, because the scaffold’s internal structure minimizes the convective flow across the membrane. Two phenomena affect the nutrient transfer process including the reaction and diffusion processes. The cell glucose consumption rate per cell, *k*_0_, can take various form of kinetics such as constant (zero-order) [26], linearly dependent on concentration (first-order) [27], or depending on *C*^2^ (second-order) [28]. According to Abdullah et al. [5], the cell seeding density is set as constant for the entire ECS region and glucose consumption rate per cell follows the first order kinetics (Equations (10)–(12)), all the used dimensional parameters are represented in Table 2.

(10) $$D_{s}\left(\frac{1}{r}\frac{\partial }{\partial r}\left(r\frac{\partial c}{\partial r}\right)+\frac{\partial^{2}c}{\partial z^{2}}\right)=k_{1}nc\;\;\left(a+m<r<A\right)$$

with the boundary conditions:

(11) $$\frac{\partial c}{\partial z}=0\;\;\;\;\;\;\;\;\;\;\;\;\;\;\;\;\;\;\;\;\;\;\;\;\;\;\mathrm{at}\;\;z=0,\;\;\;0<r<A$$

(12) $$\frac{\partial c}{\partial z}=0\;\;\;\;\;\;\;\;\;\;\;\;\;\;\;\;\;\;\;\;\;\;\;\;\;\;\mathrm{at}\;\;z=l,\;\;\;0<r<A$$

#### 2.4.2. Non-Dimensionalisation of Equations

**Figure 4:**
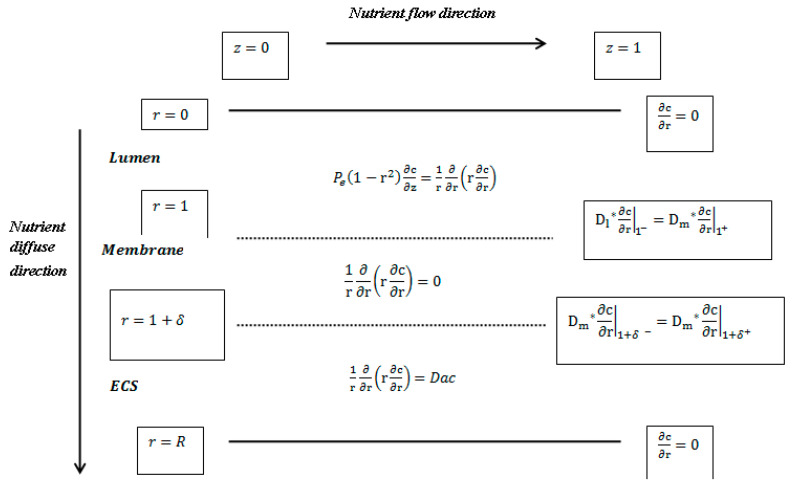
Dimensionless governing equations and boundary conditions in lumen, membrane, and extracapillary space (ECS) regions (adopted from [29]).

Non-dimensionalisation is very helpful in finding a group of parameters that are needed for the modelling process [29]. To reduce the number of parameters and simplify the model, the glucose transport equations are defined in terms of non-dimensionalised parameters with the parameters shown in Table 3. In addition to Table 3, the demonstration diagram for the dimensionless governing glucose transport equations and boundary conditions for the first-order cell kinetics in lumen, membrane, and ECS regions is shown in Figure 4. 

## 3. Results and Discussion

### 3.1. Characterisation of Materials

**Figure 5:**
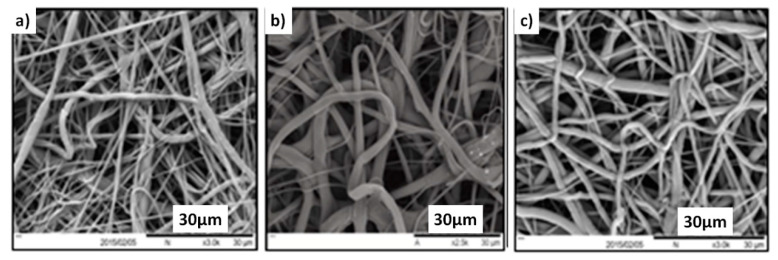
Scanning electron microscope (SEM) micrographs of the surface morphology of the electrospun scaffolds: (**a**) sample 1, (**b**) sample 2, (**c**) sample 3.

The porosity, tortuosity, fibre–fibre space, and fibre diameters were determined in this work with a view to correlate the relationships between membrane morphology and nutrient transfer. As discussed earlier, the morphology structure was observed by SEM. Figure 5 presents typical SEM images of electrospun samples 1, 2, and 3 (described in Table 1). It was observed that changing some of the key electrospinning parameters affected the fibre diameter, distribution, and pore configuration of the prepared scaffolds. For example, the feeding rate had a significant effect on the properties of the prepared scaffold, while the electrospinning time has an insignificant effect (see results for samples 1 and 3, Table 4). It was also observed that different fibre preparation (soaking time) had a non-considerable influence on the corresponding porosities. The obtained fibre–fibre space and fibre diameter for sample 1 after three measurements, as shown in Table 4, confirms the reproducibility of these scaffolds using the same conditions. The change of electrospinning processing parameters affects the final microstructural properties of the produced scaffold. As is well known, in spite of the fact that electrospinning procedures can produce consistent electrospun nanofibres from different polymers, it is difficult to control the microstructure and morphology of the resultant nanofibrous scaffold. The morphology of the last electrospun fibre is subject to the settings of the electrospinning process [30,31].

### 3.2. Fibre–Fibre Space and Fibre Diameter

The fibre–fibre distances and fibre diameters are important in determining the microstructures of the scaffold and the membrane and, thus, the nutrient transport through them. These were evaluated by SEM and calculated using ImageJ software, and the experimental results of these parameters are summarized in Table 4. 

**Figure 6:**
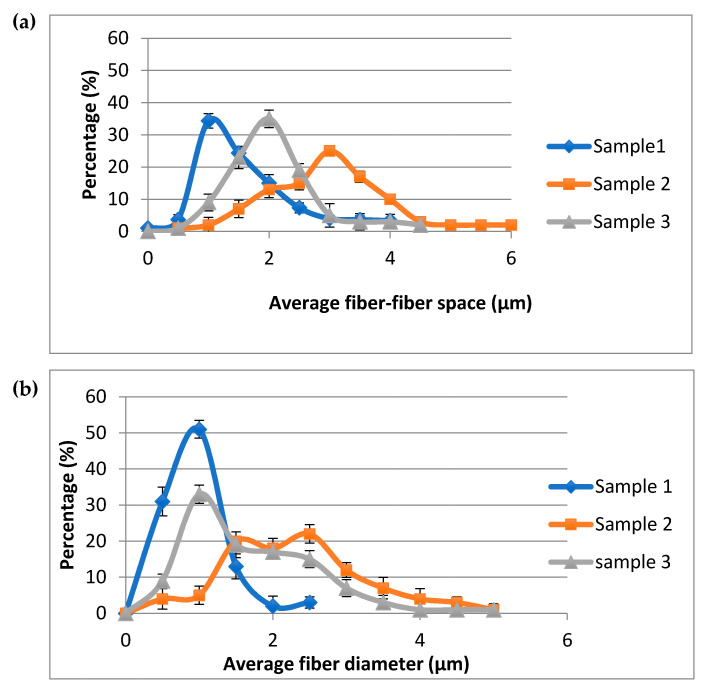
(**a**) Distribution of average fibre–fibre space and (**b**) average fibre diameter of three electrospun scaffolds (Table 1).

In the table, it shows that the fibre–fibre space and fibre diameter were similar for sample 1 for all the measures that were carried out for sample 1 (1-1, 1-2, and 1-3). This also confirmed the reproducibility and repeatability of the system. Moreover, Figure 6a shows the distribution of average fibre–fibre space and Figure 6b shows the fibre diameter for all fabricated samples. From the results, it can be seen that most were depicted as positive skewness distribution or normal distribution. Most of the distribution of both fibre–fibre distance and fibre diameter was relatively homogeneous (narrow range) with low standard deviation. From the SEM images in Figure 5, the fibre size of sample 1 (a) was obviously narrower but more homogeneous than those in sample 2 (b), which is in accordance with the results in Table 4. The microstructure of the nanofibre scaffold can impact cell diffusion [32,33,34]. Furthermore, it was reported that the fibre width and final porosity of the nanofibrous scaffolds could control the immune response for these structures [35]. In addition, it was confirmed earlier that the finer diameter of the nanofibrous electrospun scaffold (˂1 μm) would induce the minimum inflammatory response [36].

It is worth highlighting that the cell growth through nano- or microfibres are greatly influenced by the distance between fibres with relation to their diameter [37]. Furthermore, the cell propagation and the length of, was unequivocally identified with the pore size. On account of the nanofibres, the pore size was in the submicron level, constrained by the distance across the nanofibres [38]. The microfibres gave enough space for cell infiltration into the 3D fibrous scaffold; fibrous scaffolds have a little fibre width and spaces between the filaments, just as the 2D structures. We expected an entrance of cells into the electrospun framework with microfibres, as recently detailed for the PLGA frameworks [39].

### 3.3. Porosity and Tortuosity

As SEM can only show the surface morphology of thin, porous samples from the top view, three-dimensional porosity and tortuosity were found to be difficult to determine reliably in this case. The measured porosity of all prepared electrospun scaffolds with different soaking time is shown in Table 5. In terms of the porosity values, the results did not show significant changes with changes in the time of soaking. Based on the low standard deviations recorded for all the measured samples and given that the porosity values for each sample were relatively the same during different soaking times, therefore, in this study, porosity was assumed to be constant during the diffusion experiments, the effects of which on glucose diffusivity were ignored.

### 3.4. Experimental Values Glucose Diffusivities in Different Electrospun Scaffolds

**Figure 7:**
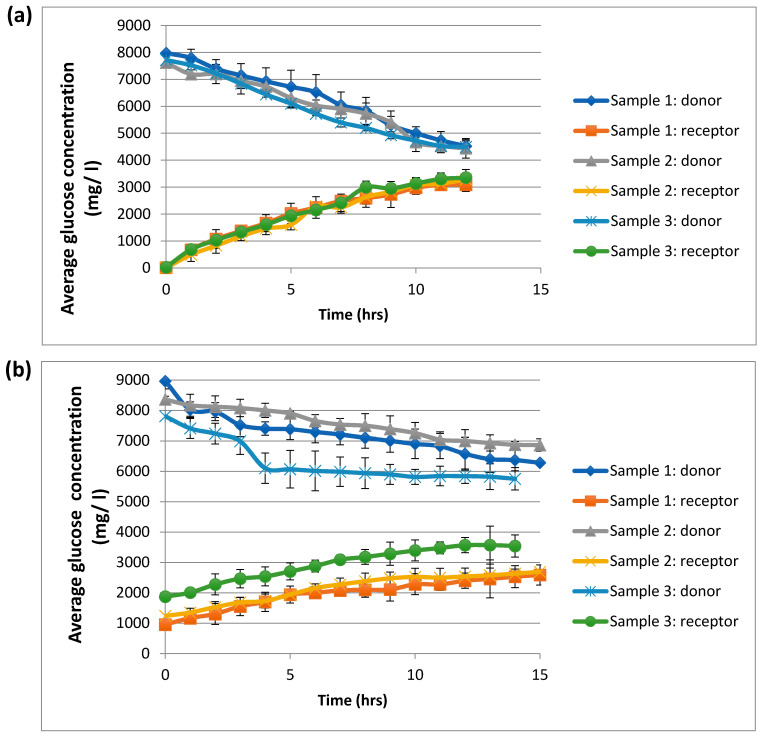
Average glucose concentration change versus time through electrospun scaffolds in (**a**) water and (**b**) CCM. Sample measurement was repeated three times.

With the same electrospun scaffolds, the glucose diffusion experiments were carried out to study the glucose diffusion process in water and CCM. Figure 7 shows the trend of glucose concentration changes for both donor and receiver chambers in both water (Figure 7a) and CCM (Figure 7b). From observing the behaviour of the glucose concentration changes in Figure 7, it can be noted that the glucose concentration decreased in the donor phase and increased in the receiver phase. This confirms that the glucose molecules diffuse from a high concentration to relatively low concentration location until it reaches the equilibrium state. This can be related to the concentration difference between these two phases that led to weaker driving force for the glucose molecules. The utilized membranous scaffolds are supposed to act as resistance for the glucose diffusion; however, glucose can still diffuse through membranes and scaffolds, which means that these membranes possess a lower diffusion coefficient.

From the previous obtained results for distance between fibres, both effective diffusivities in water and CCM were summarized in Table 6, to be used in the calibration of the image processing tool and can also be applied in the mathematical modelling. From the table, as expected, effective diffusivities showed an increase at a larger fibre–fibre space, indicating the least resistance to diffusion through the pores in both water and CCM. This could also be noted from Figure 7 that the diffusivities in both water and CCM were slightly increased with the increasing porosity. As the pore size increased, the diffusivity also increased. It was also found that the diffusion coefficients in CCM were smaller than those in water. The results are in accordance with the early reported work by Suhaimi et al. [18]. 

### 3.5. Image Analysis for Commercial Membranes and In-House Electrospun Scaffolds

**Figure 8:**
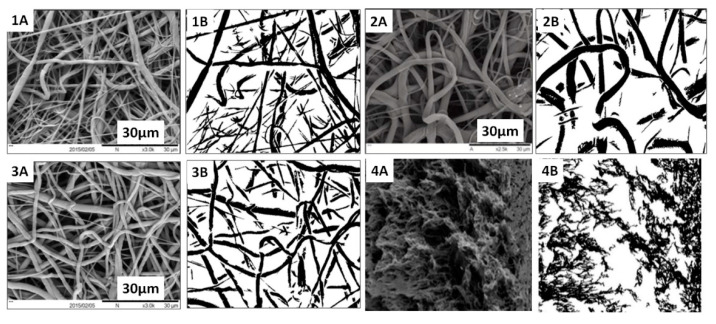
Typical original and black and white SEM images for (**1A**,**1B**) sample 1, (**2A**,**2B**) sample 2, (**3A**,**3B**) sample 3 and (**4A**,**4B**) polyvinlyidene fluoride (PVDF) membrane (commercial membrane).

**Figure 9:**
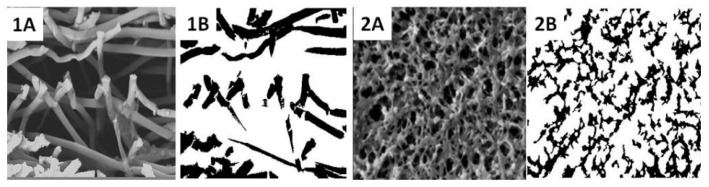
Typical original and black and white cross-section-view SEM images for (**1A**,**1B**) sample 1 compared to the (**2A**,**2B**) PVDF membrane.

In order to build a more accurate image processing tool, aside from the three in-house electrospun scaffolds (Table 1), polyvinlyidene fluoride (PVDF) commercial membranes were employed in this study to calibrate this method. This provided the confidence that the methodology is applicable to a range of porous microstructures. Multiple SEM images of all scaffolds were used, and the porosity value was calculated for each image. In addition, the effective glucose diffusivities in water and CCM were determined using the same method. The final value was the average obtained from each of the original images and processed black and white top-view images (Figure 8) and cross-section-view images for sample 1 compared to the PVDF membrane (Figure 9).

#### 3.5.1. Porosities and Tortuosity Analyses

**Figure 10:**
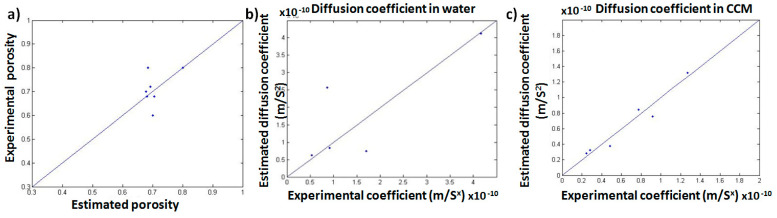
Scatter plots comparing the experimental and estimated results for (**a**) porosity, (**b**) diffusion coefficient in water, and (**c**) diffusion coefficient in CCM.

It can be seen from the scatter plot (Figure 10a) that the obtained experimental values were consistent with those estimated via the image analysis (five values out of seven estimated values were almost equal to the experimental values). This provides the confidence in the applicability of the used method to calculate the porosity of a material with unknown porosity or for samples subjected to biological conditions (during in vitro or in vivo conditions) as it is difficult to determine the porosity during such condition. This could provide a good evaluation tool to estimate the porosity during the infiltration of cells and their growth within the scaffold. Moreover, tortuosity was determined using the same analysis technique and the estimated results for the prepared scaffolds were compared with their corresponding experimental results, as listed in Table 7. This comparison revealed that the estimated results were relatively lower than those recorded by the experimental work. This was expected, as in the simulation process, the shortest path was chosen for the purpose of analysis, which resulted in lower tortuosity values.

#### 3.5.2. Effective Diffusion Coefficient in Water and CCM

Figure 10b,c shows the scatter plot of the estimated effective diffusivities in porous samples against the experimental results in water and CCM, respectively. An effective diffusion coefficient in water produced values close to those experimentally obtained. Out of the five points, three points matched well, as can be observed from the scatter plot (Figure 10b). Thus, the method for calibration and calculation of diffusion coefficient can be used to predict the diffusion coefficient value for the unknown samples. Figure 10c shows that the estimated values (after the calibration) were similar to the experimentally obtained values for CCM. This implies that the code used for the simulation of an effective diffusion coefficient can be relied upon for predicting the diffusion coefficient of a material with unknown diffusion coefficient or for samples subjected to biological conditions (during in vitro or in vivo conditions) as it is difficult to determine the diffusion coefficient during such conditions. The estimated and experimental results are demonstrated in Table 8.

### 3.6. Modelling Nutrient Transport Taking into Consideration of the Microstructure of the Scaffold

Estimated results from an image processing tool were used in this study to observe the effect of the diffusion coefficient on the nutrient transfer process in HFMB. The variables were all converted into non-dimensional groups based on the equations in Table 3. Except for the scaffold parameter (normalized scaffold (PCL) diffusivity), other parameters including the HFMB dimensional parameter, membrane material, and processing parameters were considered as constants and are listed in Table 9. Undoubtedly, the understanding of glucose transport on a very basic level is significant in forming the structure of the HFMB. Several studies and numerical models have been utilized to explore the diffusion of glucose within the HFMB [40,41,42,43]. Nevertheless, it remains uncommon to relate the dispersive impacts that happen when glucose spreads inside a stream field to aa simulated one.

**Figure 11:**
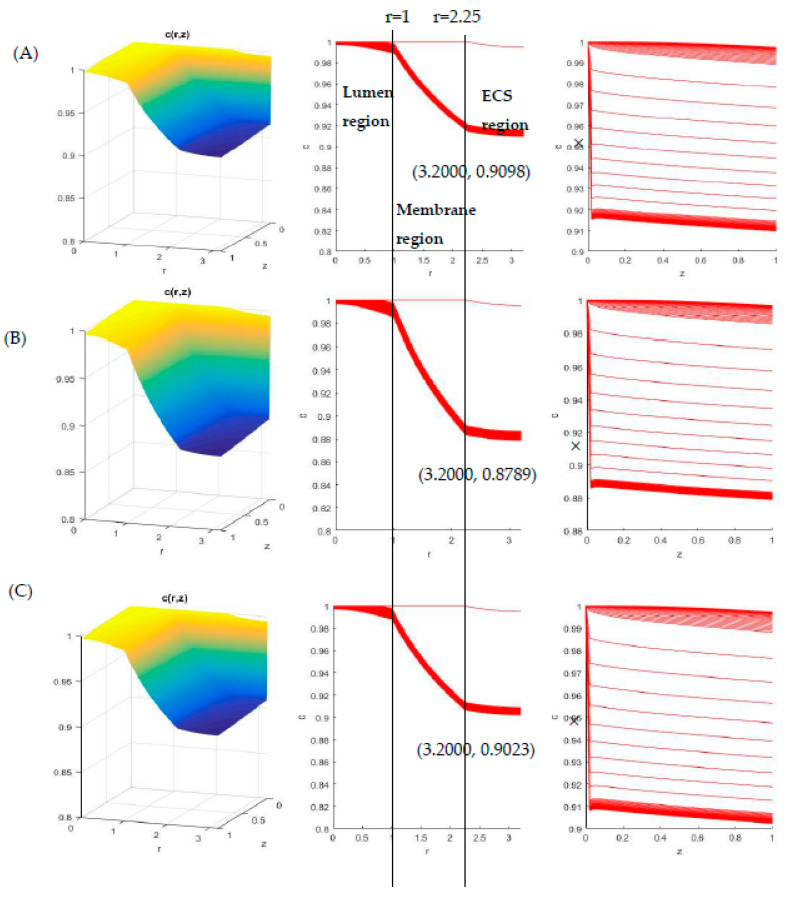
Glucose concentration profiles in CCM at 37 °C with (**A**) PVDF-PCL sample 1(**B**) PVDF-PCL sample 2, and (**C**) PVDF-PCL sample 3.

As an example of numerical simulation results, Figure 11 shows the glucose concentration profile for the combination of PVDF-PCL membrane-scaffold HFMB system with CCM at 37 °C. With the consumption of cells and diffusion, the glucose concentration has a significant reduction radially and axially. Additionally, it can be seen that the glucose concentration changes were more obvious along the radius than along the axial direction of the bioreactor. In the meantime, data were more dispersed along the axial direction. It thus indicates that the diffusion process is the main factor of the glucose concentration change. With r = a = 1 and r = a + δ = 2.25 in Figure 11, very minor changes were exhibited in lumen and ECS region. However, between r = 1 to r = 2.25, the concentration dropped steeply, which proves that the diffusion across the PVDF membrane is the limiting parameter in HFMB.

**Figure 12:**
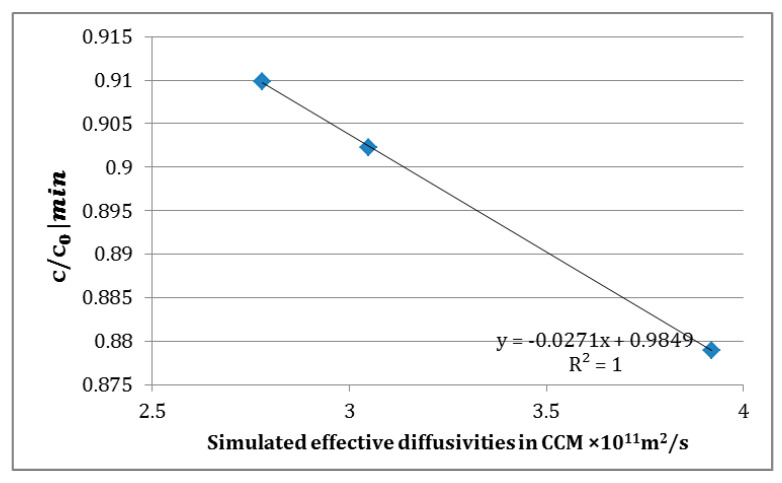
Relationship between simulated effective diffusivity and minimum concentration in HFMB.

The minimum value of concentration was found near the outlet of the system (z = 1, r = R = 3.2), which is marked with the value in Figure 11. The relationship with the simulated effective diffusivities is shown in Figure 12. Although the main diffusion process occurs in the membrane region, the choice of membranes (i.e., their pore morphologies) affects the minimum glucose concentration in the HFMB. The minimum glucose concentration near the outlet of HFMB is inversely proportional to the simulated effective diffusivities in CCM. In other words, due to the same inlet concentration, the glucose consumption efficiency is improved with increasing diffusivity. However, the cell density is highly nutrient concentration dependent. In order to culture the higher density of cells, the suitability of the chosen scaffold is critical, which should be considered in the HFMB design.

The roles of a dimensionless group of variables such as Pe and Da in determining the nutrient transport behaviour in the HFMB has been discussed in length in our earlier paper [29]. As the glucose transport in the lumen region of HFMB is governed by the Péclet number, varying this dimensionless number corresponds to varying the length of the bioreactor, or vice versa, provided all other factors remain the same. Consistent with [30], we observed here that the minimal glucose concentration decreased with an increase in the length of the bioreactor as the Pe number decreased (Pe = 20, Pe = 8.06 and Pe = 1). This can be attributed to the increase in the diffusion distance of glucose due to the increase in the bioreactor length. The results in this paper demonstrated similar trends when compared to [29] for a similar combination of porous materials and therefore are not shown in this paper. However, it suffices to note that the bioreactor length should be considered carefully, keeping in mind the glucose transfer limitation while developing a larger bioreactor scale, especially for a long-term culture process within the scaffold region. 

As mentioned earlier, the present model is based on the Krogh cylinder model and it is defined that HFMB consists of numerous identical hollow fibres. Varying the dimensionless Krogh cylinder radius (R) physically corresponds to the changes in the spacing between the fibres. This effect is important due to its potential in limiting the glucose concentration, which is essential for cell growth in the scaffold region. In agreement with [29], the current work also showed that the lowest concentration will occur at the largest value of *R*. This is due to an increase in diffusion distance from the inlet (*c* = 1) toward the outlet of the bioreactor.

## 4. Conclusions

Determining the nutrient diffusion mechanism in a bioreactor is very important for cell vascularization to take place, especially in BTE due to the lack of blood vessel networks (in vitro). The combined application of image processing tool (ImageJ software) and modelling of HFMB makes it possible to monitor the diffusion process in the bioreactor, as long as the SEM images of scaffolds (PCL electrospun nanofibrous scaffold in this study) are available. Moreover, the processed images were used to estimate porosity and glucose diffusivity in water and CCM with MATLAB codes that were compared with those determined via diffusion cell experiments. It can be seen that results obtained from image processing are in good agreement to the experimentally obtained results with regard to both the porosity and diffusion coefficient. Hence, the mathematical code derived from this study was proven to be reliable and could be applied to simulate the glucose concentration change. The results showed that the diffusion process is the limiting step of nutrient transfer in HFMB. Increasing the fibre spacing and bioreactor length varies the minimal glucose concentration near the bioreactor outlet (*z* = 1). With increasing glucose diffusivity across the PCL scaffold, the minimum concentration (at *z* = 1) decreased in a linear relationship. Although the efficiency of glucose consumption improved, low glucose concentration may decrease the cell density. Therefore, it was concluded that these parameters should be considered when developing a large-scale bioreactor for producing 3D bone tissues. This study successfully developed a possible simulation method that could be implemented in the assessment of the concentration change process of the main nutrient (e.g., glucose) for cell growth, with only known SEM images. Thus, it will be a huge help to design better HMFB with reduced experimental work and time.

## Figures and Tables

**Table 1:** Electrospinning process parameters used in the fabrication of in-house membranes and scaffolds.

Porous Sample No	Polymer Flow Rate (mL/h)	Electrospinning Duration (min)	Number of Syringes
**1**	1	90	1
**2**	2	90	1
**3**	1	45	2

**Table 2:** Dimensional parameters and their values used in this study.

Parameters	Values	Units	References
Fibre inner radius (a)	1.0 × 10^−4^	m	[26]
Fibre membrane (PVDF) thickness (m)	1.25 × 10^−4^	m	[18]
Fibre length (l)	3.0 × 10^−2^	m	[26]
Krogh cylinder radius (A)	3.2 × 10^−4^	m	[26]
Average axial flow velocity (U_0_)	0.745 × 10^−2^	m/s	[26]
CCM kinematic viscosity (ʋ)	8.28	m^2^/s	[17]
Temperature	37	°C	N.A.
Diffusivity of glucose in lumen (D_l_)	5.67 × 10^−10^	m^2^/s	[17,19]
Diffusivity of glucose in membrane (D_m, PVDF_)	7.68 × 10^−10^	m^2^/s	[18,19]
Diffusivity of glucose in ECS (D_s_)	…	m^2^/s	…
Glucose inlet concentration (C_0_)	5.55	mol/m^3^	[26]
Cell seeding density (n)	2.0 × 10^12^	cells/m^3^	[26]
Glucose consumption rate per cell (k_0_)	3.83 × 10^−16^	mole/cell.s	[26]
Glucose consumption rate coefficient (nk_1_ = nk_0_/C_0_)	1.38 × 10^−4^	s^−1^	N.A.
Glucose degradation rate in the water/acidic environment	~10^−5^–10^−4^	s^−1^	[29]

**Table 3:** Non-dimensional parameters and conversion equations.

Parameters	Dimensionless Equation
Fibre lumen aspect ratio	$\epsilon =\frac{a}{l}$
Dimensionless membrane thickness	$\delta =\frac{m}{a}$
Dimensionless Krogh cylinder radius	$R=\frac{A}{a}$
Normalized membrane (PVDF) diffusivity	$D_{m}*=\frac{D_{m}}{D_{l}}$
Normalized membrane (PVDF) diffusivity	$D_{s}*=\frac{D_{s}}{D_{l}}$
Péclet number	$\mathrm{Pe}=\frac{2U_{0}l}{D_{l}}\frac{a^{2}}{l^{2}}$
Damköhler number	$\mathrm{Da}=\frac{k_{1}na^{2}}{D_{s}}$
Reynolds number	$Re=\frac{U_{0}a}{ʋ}$

**Table 4:** Average fibre-fibre space and fibre diameter of electrospun scaffolds.

Sample No.	Average Fibre–Fibre Space (μm)	Average Fibre Diameter (μm)
**1-1**	1.38 ± 0.75	0.78 ± 0.41
**1-2**	1.43 ± 0.53	0.88 ± 0.18
**1-3**	1.48 ± 0.36	0.78 ± 0.36
**2**	3.80 ± 1.69	2.10 ± 0.77
**3**	1.88 ± 0.77	0.91 ± 0.64

**Table 5:** The mean values of porosity and tortuosity at different soaking times in water.

Sample No. (Table 1)	The Time of Soaking in Water (hrs)	Mean Value of POROSITY	Calculated Tortuosity (Dimensionless)
**1**	0	0.607 ± 0.079	9.22
	4	0.611 ± 0.033	9.27
	8	0.619 ± 0.043	9.39
	12	0.611 ± 0.014	9.27
**2**	0	0.741 ± 0.052	8.58
	4	0.758 ± 0.048	8.78
	8	0.725 ± 0.032	8.40
	12	0.736 ± 0.015	8.52
**3**	0	0.685 ± 0.007	9.01
	4	0.720 ± 0.005	9.47
	8	0.726 ± 0.010	9.55
	12	0.696 ± 0.007	9.16

**Table 6:** Effective glucose diffusivities through membrane in water and CCM at 37 °C.

Sample No.	Effective Diffusivities in CCM × 10^11^ m^2^/s	Effective Diffusivities in Water × 10^11^ m^2^/s
**1**	2.83 ± 0.12	6.31 ± 0.31
**2**	3.75 ± 0.27	8.27 ± 0.23
**3**	3.22 ± 0.11	7.38 ± 0.27

**Table 7:** Calibration for estimated and experimental tortuosity at 37 °C.

Electrospun Scaffold	Simulated Tortuosity (Surface Values)	Experimental Tortuosity (3D Values)
**Commercial PCL**	1.56 ± 0.015	2.5
**1**	1.81 ± 0.026	9.22
**2**	2.47 ± 0.030	8.59
**3**	1.55 ± 0.021	9.01

**Table 8:** Estimated and experimental effective diffusivities of selected scaffolds at 37 °C.

Sample No.	Effective Diffusivities of Scaffold in CCM × 10^11^ m^2^/s		Effective Diffusivities of Scaffold in Water × 10^11^ m^2^/s	
	Estimated via Image Processing	Experimental	Estimated via Image Processing	Experimental
**1**	2.78	2.83 ± 0.12	5.72	6.31 ± 0.31
**2**	3.92	3.75 ± 0.27	8.69	8.27 ± 0.23
**3**	3.05	3.22 ± 0.11	7.57	7.38 ± 0.27

**Table 9:** Dimensionless variable used in mathematical modelling.

Parameters		Values	Symbols
Fibre lumen radius		1.00	A
Fibre lumen aspect ratio		3.33 × 10^−3^	ϵ
Dimensionless membrane thickness		1.25	δ
Dimensionless Krogh cylinder radius		3.2	R
Normalized membrane (PVDF) diffusivity		0.125	D_m_*
Normalized scaffold (PCL) diffusivity	Sample 1	0.49	D_s_*
	Sample 2	0.69	
	Sample3	0.54	
Péclet number		8.06	Pe
Damköhler number		9.93 × 10^−3^	Da
Reynolds number		0.996	Re

## Data Availability

For any additional data, please contact the corresponding author Dr Das via email.
